# Editorial: Crime, mental health, and the law: A psycho-criminological perspective

**DOI:** 10.3389/fpsyg.2023.1142596

**Published:** 2023-02-23

**Authors:** Heng Choon (Oliver) Chan

**Affiliations:** Department of Social Policy, Sociology, and Criminology, School of Social Policy, University of Birmingham, Birmingham, United Kingdom

**Keywords:** psycho-criminology, crime, mental health, law, psycho-criminological perspective

Broadly speaking, psychology is the scientific study of behavior and mental processes, while criminology is the scientific study of crime and criminal offenders. Psychological criminology (or simply as psycho-criminology), which combines the two, is generally concerned with the use of psychological knowledge and skills to explain, describe, and potentially prevent or deal with deviant and criminal behavior (Hollin, 2012; Chan and Ho, 2017; Chan and Sheridan, 2020; Chan and Adjorlolo, 2021; Chan and Wong, 2023). More specifically, psycho-criminology (an abbreviated term for “psychological criminology”) is the study of individual criminal behavior, with particular interest on how the behavior is learned, evoked, continued, and evolved as a result of personality, social, and/or environmental influences (Bartol, 2002). In the words of Wortley (2011), psychological criminology addresses the general question, “What is it about the individuals and their experiences that cause them to commit crime and/or to become criminal?” (p. 1).

In this Research Topic (RT), the focus lies on the application of psycho-criminological approaches and constructs to crime, criminal and civil law, and the influence of law on mental health and behavior. This RT aims to advance our understanding of psycho-criminological mechanisms (i.e., personal, social, and environmental influences) associated with different criminal behavior in the intersections of mental health and the law. The 10 articles included in this RT explore varied aspects of crime and criminal behavior through the application of psychological concepts and theories to increase our understanding of crime, delinquents and criminal offenders, and their behavior. The highlight of this RT is the range of contribution conducted with six sampling populations, namely Spanish, mainland Chinese, Hong Kongers, Americans, Germans, and Italians. Besides, the collection of these articles addresses the different aspects in the criminal justice system (e.g., youth, correctional, legal, and mental health) from a psycho-criminological standpoint.

This RT begins with an empirical study of testing a typology of Spanish homicides by Pecino-Latorre et al.. Using a sample of 448 homicides, this article examines the effectiveness and validity of the Action System model to distinguish thematically between the structure of the homicides and to generate a homicide typology in Spain based on the relationships between the modus operandi, and victim and offender characteristics. Four homicide typologies were identified: Expressive, Adaptive, Integrative, and Conservative. Next, Zhu and Shek investigate the effect of individual dimensions and the global positive youth development (PYD) measures on adolescent delinquency, individually; and the underlying mediating effect of life satisfaction. Employing a two-wave longitudinal data collected from 2,648 mainland Chinese adolescents, findings are found to be consistent with the general theoretical prediction of the PYD approach; in which different PYD attributes (cognitive-behavioral competence, prosocial attribute, positive identity, and general PYD attribute) are inversely related to concurrent and future adolescent delinquency. Moreover, the negative predictions are mediated by the adolescents' life satisfaction. Also recruiting mainland Chinese adolescents, Xiong et al. construct a moderated mediation model to test the mechanisms underlying the relationship between perceived discrimination and proactive and reactive aggression in a longitudinal data of 470 mainland Chinese migrant students (aged 11–17). They observed that perceived discrimination fosters negative emotions, which in turns increase reactive aggression. Furthermore, socioemotional support reduces the negative impact of perceived discrimination on reactive aggression by weakening the relationship between perceived discrimination and negative emotions.

In Hong Kong, Chan sampled 1,171 young adults (aged 18–40) to explore the psychosocial risk factors of risky sexual behavior (RSB) by testing the theoretical propositions of several criminological theories (the theories of self-control, general strain, social learning, social control, and routine activity). Relative to females, males possess significantly higher mean levels of general, penetrative, and non-penetrative RSB; and negative temperament, use of alcohol and other drugs, and paraphilic interests. Males and females are generally sharing a similar set of psychosocial risk factors (use of alcohol and other drugs, and paraphilic interests) for their involvement in general, penetrative, and non-penetrative RSB. Similarly exploring risky behaviors, Méndez et al. explore the different adaptation profiles (personal, school, and social) in adolescents based on their interpersonal risk factors on drug use. Analyzing 1,201 secondary school students (aged 11–18 years) in Spain, a latent class analysis generated three different types of adaptation: Maladjusted group, At-risk group, and Adjusted group.

Next, Acklin and Velasquez argue that Structured Professional Judgment (SPJ) methods can be a corrective approach for unstructured clinical judgment that prone to evaluator bias and suboptimal levels of inter-rater reliability. The authors propose a SPJ model for criminal responsibility evaluations translated from violence risk assessment methodology. Sampling 230 patients in 13 forensic psychiatric hospitals in Germany, Büsselmann et al. measure the patients' quality of life in forensic psychiatric hospital using the Measuring the Quality of Prison Life (MQPL) questionnaire. They found that the adapted MQPL questionnaire demonstrates good internal reliability and construct validity. The next article by Titze et al. examine the self-reported acculturation processes and associated individual and social factors in a similar sampling population of 235 forensic psychiatric patients with a migration background in 11 forensic hospitals in Germany. The findings indicate that the patients oriented themselves more toward the culture of admission and less toward the country of origin than the reference sample did.

In Italy, Rossetto et al. retrospectively compared 42 readmitted with 48 non-readmitted females in an Italian forensic psychiatric hospital through a minimum of 42 months follow-up (ranges from 3.5 to 10 years). Their findings indicate that readmitted females were positively associated with the presence of substance use disorders and a primary diagnosis on Axis II. The final article of this RT is authored by Brown et al.. They examine the fetal alcohol spectrum disorder (FASD) by proposing a renewed focus on applying and adapting the Risk-Need-Responsivity (RNR) approach to individuals with FASD in criminal justice settings. The authors argue that the use of RNR approach can better determining the needs and interventions in reducing the propensity of offender recidivism.

With studies conducted in Spain, Mainland China, Hong Kong, the USA, Germany, and Italy, the 10 articles in this RT collectively demonstrate the importance of applying psycho-criminological knowledge and skills to better understand the underlying mechanism (i.e., personal, social, and environmental influences) associated with different criminal behavior in the intersections of mental health and the law. Having studies from different cultures and jurisdictions have clearly demonstrated that a combined etic-emic approach is arguably more appropriate when studying crime and criminal behavior, and developing culturally sensitive assessments and interventions (Ho and Cheung, 2007).^1^ It is important to continue with more international research to advance our knowledge of research and best practice that have implications for further research, practice, and policy development/refinement in this emerging field of psycho-criminology.

## Author contributions

The author confirms being the sole contributor of this work and has approved it for publication.

## Footnotes

^1^The etic approach proposes that theories and practices are universal and can be transferred to other cultures with little modification, while the emic approach assumes that behavior is culture-specific. The combined etic-emic approach suggests that elements (e.g., theoretical concepts and models) that appear to be universal can be initially identified and other emic elements (e.g., strategies and practices) can be developed based on specific cultural and population characteristics.

## References

[B1] Bartol C. R. (2002). Criminal Behaviour: A Psychosocial Approach, 6th Edn. Upper Saddle River, NJ: Prentice Hall.

[B2] Chan H. C. O. Adjorlolo S. (2021). Crime, Mental Health and the Criminal Justice System in Africa: A Psycho-Criminological Perspective. Cham, Switzerland: Palgrave Macmillan. 10.1007/978-3-030-71024-8

[B3] Chan H. C. O. Ho S. M. Y. (2017). Psycho-Criminological Perspective of Criminal Justice in Asia: Research and Practices in Hong Kong, Singapore, and Beyond. Oxfordshire, UK: Routledge.

[B4] Chan H. C. O. Sheridan L. (2020). Psycho-Criminological Approaches to Stalking Behavior: An International Perspective. West Sussex, UK: John Wiley and Sons.

[B5] Chan H. C. O. Wong R. W. Y. (2023). Animal Abuse and Interpersonal Violence: A Psycho-Criminological Understanding. West Sussex, UK: John Wiley and Sons.

[B6] Ho S. M. Y. Cheung M. W. L. (2007). “Using the combined etic-emic approach to develop a measurement of interpersonal subjective well-being in Chinese populations,” in Oxford Handbook of Methods in Positive Psychology, eds A. D. Ong and M. van Dulmen pp. (New York, NY: Oxford University Press), 139–152.

[B7] Hollin C. R. (2012). “Criminological psychology,” in The Oxford Handbook of Criminology, 5th Edn., eds M. Maguire, R. Morgan, and R. Reiner (New York, NY: Oxford University Press), 81–113.

[B8] Wortley R. (2011). Psychological Criminology: An Integrative Approach. Oxon, UK: Routledge. 10.4324/9780203806098

